# Evaluation of tobacco specific nitrosamines exposure by quantification of 4-(methylnitrosamino)-1-(3-pyridyl)-1-butanone (NNK) in human hair of non-smokers

**DOI:** 10.1038/srep25043

**Published:** 2016-04-26

**Authors:** Raúl Pérez-Ortuño, Jose M. Martínez-Sánchez, Marcela Fu, Esteve Fernández, José A. Pascual

**Affiliations:** 1 Group of integrative pharmacology and systems neuroscience, Programme of Neurosciences, IMIM (Hospital del Mar Medical Research Institute). Parc de Recerca Biomèdica de Barcelona, Doctor Aiguader, 88, 08003 Barcelona, Spain; 2 Department of Experimental and Health Sciences, Universitat Pompeu Fabra. Parc de Recerca Biomèdica de Barcelona, Doctor Aiguader, 88, 08003 Barcelona, Spain; 3 Tobacco Control Unit, Cancer Control and Prevention Programme, Institut Català d’Oncologia-ICO. Av. Granvia de L’Hospitalet 199-203, 08908 L’Hospitalet de Llobregat (Barcelona), Spain; 4 Cancer Control and Prevention Group, Institut d’Investigació Biomèdica de Bellvitge-IDIBELL. Av Granvia de L’Hospitalet 199-203, 08908 L’Hospitalet de Llobregat (Barcelona), Spain; 5 Biostatistic Unit, Department of Basic Science, School of Medicine and Health Sciences, Universitat Internacional de Catalunya. Carrer Josep Trueta s/n. 08915 Sant Cugat del Valles (Barcelona), Spain; 6 Department of Clinical Sciences, School of Medicine, Universitat de Barcelona. Carretera de la Feixa llarga s/n. 08908 L’Hospitalet del Llobregat (Barcelona), Spain

## Abstract

Chronic exposure to specific carcinogens present in secondhand smoke has been associated with different types of cancers. Hair is an ideal matrix to develop a proper biomarker as it absorbs substances in circulation and allows measuring their average concentration over long periods of time. A method was developed for the simultaneous quantification of nicotine, cotinine, NNN, NNK and NNAL in 20 mg human hair samples. Concentrations were significantly different depending on the declared exposure. This study shows for the first time that NNK is present in hair samples from non-smokers in concentrations much higher than any other tobacco specific nitrosamine. NNN could also be detected in samples from the most exposed non-smokers while, as previously reported, NNAL was undetectable. NNK correlates well with nicotine and cotinine (rsp = 0.774 and rsp = 0.792 respectively, p < 0.001 in both cases). However, NNN concentrations did not correlate with any of the other analytes. Ratios between NNK and nicotine show variability with different concentrations of NNK present in samples with similar nicotine values. NNK has proven to be the best marker of tobacco specific nitrosamines in hair. Monitoring NNK may provide a good estimation of cancer risk associated with exposure to secondhand smoke.

Exposure to secondhand tobacco smoke (SHS) increases the risk of respiratory and cardiovascular diseases^1^,^2^ and it is classified as Group1 carcinogen in humans by the International Agency for Research on Cancer (IARC)^1^. Tobacco specific nitrosamines (TSNAs) and particularly N′-nitrosonornicotine (NNN) and 4-(methylnitrosamino)-1-(3-pyridyl)-1-butanone (NNK), are also classified as Group 1 carcinogens in humans^3^ (Fig. 1). While NNN is strongly associated with esophageal cancer risk in smokers^4^,^5^,^6^,^7^, NNK and its main metabolite 4-(methylnitrosamino)-1-(3-pyridyl)-1-butanone (NNAL) are involved in lung, pancreas and other sorts of cancers^4^,^5^,^8^,^9^,^10^,^11^,^12^,^13^.

Moreover, NNN and NNK are the most prevalent strong carcinogens in unburned tobacco products^4^. They are present in mainstream and secondhand smoke^14^,^15^ but also in thirdhand smoke^16^,^17^. Whereas airborne nicotine concentrations decrease over time, NNK continues being generated in smoke even hours after it was emitted^18^. Consequently, second and thirdhand smoke may lead to a significant unnoticed uptake of NNK.

Monitoring chronic exposure to tobacco specific carcinogens (i.e. TSNAs), particularly in non-smokers, is very relevant in evaluating the impact on public health of the implementation of smoke-free laws. Biomarkers of tobacco smoke exposure are essential to study the metabolic fate of tobacco products and their potential health risk. Nicotine and its main metabolites cotinine, and *trans*-3′-hydroxycotinine have been extensively used as specific markers in smokers and non-smokers exposed to SHS^19^,^20^,^21^,^22^,^23^,^24^,^25^,^26^. TSNAs occur in much lower concentrations and their detection is challenging^27^,^28^,^29^. Exposure to TSNAs has been mainly approached by quantifying total NNAL (free, plus N and O-glucuronides) in urine of smokers and non-smokers, although serum has also been used^30^,^31^,^32^,^33^,^34^,^35^. The detection of NNAL in urine samples from non-smokers has strengthened the argument that passive smoking is linked with lung cancer^5^. NNAL/cotinine ratios are significantly higher in samples from non-smokers than from smokers, suggesting that involuntary or passive smoking, i.e. exposure to TSNAs in second and third-hand smoke, might be underestimated by using cotinine as biomarker of exposure^28^,^36^,^37^. The analysis of urine, blood or saliva gives a good indication of the exposure in the last few days, considering the half-life of the analyte and the nature of the biological matrix^9^,^30^. Conversely, analysis of keratinic matrices, such as hair and nails, has some advantages over those biological fluids. Circulating drugs are embedded in hair or nails as they grow. The adsorption mechanisms are not fully understood but, in hair, there seems to be a significant role played by melanines with basic and less polar compounds being selectively enriched^20^,^22^,^38^. Nicotine, for example, is found in hair in concentrations 10–100 times higher than its main metabolite cotinine^20^. The absence of metabolism in these solid matrices precludes degradation of the analytes and, as hair grows 1 cm per month on average, a segmental analysis allows ‘recording’ of tobacco cumulative exposure over long periods of time^39^,^40^. Exposure to SHS measured through hair analysis is therefore less affected by the daily variability allowing a more robust comparison. This is particularly relevant when dealing with a non-smoker population, irregularly exposed to SHS^27^.

NNAL has been detected in toenails and hair from regular smokers (i.e. more than 10 cigarettes per day) in concentrations ranging 0.04–4 pg/mg and 0.27–0.67 pg/mg respectively^41^,^42^. However it has been undetectable in samples from non-smokers^41^. NNN has also been found in toenails from smokers and non-smokers in concentrations below 5 pg/mg^43^.

The objective of this project was the analysis of different tobacco specific biomarkers, including particularly TSNAs, in hair samples from non-smokers in an attempt to find the best candidate. Considering the behaviour of nicotine and cotinine, the hypothesis was that NNK might be the most suitable hair biomarker of cumulative exposure to TSNAs.

## Results

A procedure was developed for the simultaneous quantification of nicotine, cotinine, NNN, NNK and NNAL in hair samples. Lower limits of quantification (LLOQs) were set at 25, 2.5, 0.25, 0.10 and 0.063 pg/mg respectively, using 20 mg of hair. The amount of sample available varies a lot depending on the subject, therefore the use of lower or higher amounts of matrix was tested and validated as not affecting precision and accuracy. However, there is an obvious impact on the limit of quantification reached for any particular sample. The analysis of sample amounts between 5 and 50 mg did not produce any significant differences in quantification other than the achievable LOQs.

Deuterated internal standards (I.S.) were used for all analytes. Linearity was proven to be good for the calibration ranges tested with coefficients of determination (r^2^) being above 0.99 in all cases. Confidence intervals (IC95%) of the regression parameters of the calibration curves prepared in matrix and in absence of matrix showed not to be significantly different (p < 0.05). Calibrators prepared in matrix complied with the accuracy requirements when back-calculated using curves prepared in absence of matrix. Precision, expressed as the coefficient of variation, and accuracy, as percent of error, was determined by quantifying five replicates of control samples prepared in hair at low, medium and high levels through the calibration range. Typical values were well below 10%. Only cotinine and NNAL at the lowest concentration gave a higher but still acceptable variation (Table 1). Extraction recoveries were found to be appropriate ranging 65–97% depending on the analyte. However, as shown in similar methods, the matrix effect significantly influences the overall process efficiency, but those effects proved to be properly compensated by the deuterated internal standards used for quantification purposes. N-Glucuronides of the deuterated analogues of nicotine, cotinine and NNAL (those commercially available) were also taken through the procedure and, as previously reported^43^,^44^,^45^ it was proven that N-glucuronides of those compounds are completely hydrolyzed under the basic incubation conditions used to digest hair. The O-glucuronide of NNAL was not completely hydrolysed, although previous publications suggest that it is not even present in hair^42^. Therefore, it can be stated that the results account for the total amount of the analytes (free plus glucuronides). Individual standards of nicotine, cotinine, 3′-hydroxycotinine and nornicotine (1 μg each) were taken through the analytical procedure and NNK production in measurable amounts was discarded. The identity of both NNK and NNN was confirmed following the chromatographic and MS criteria of the latest technical document issued by the World Anti Doping Agency (TD2015IDCR)^46^.

Hair of non-smokers (N = 48) were analysed for nicotine, cotinine, NNN, NNK and its main metabolite NNAL. From the 48 volunteers recruited, 24 declared living with at least one smoker while 24 non-smokers lived in smoke-free homes. However, all were asked about other sources of potential exposure (i.e. at work, transportation, leisure time). Only 6 declared not being exposed at any time. Exposure at home resulted in the most significant source, as measured by salivary cotinine values^19^.

**Figure 1:**
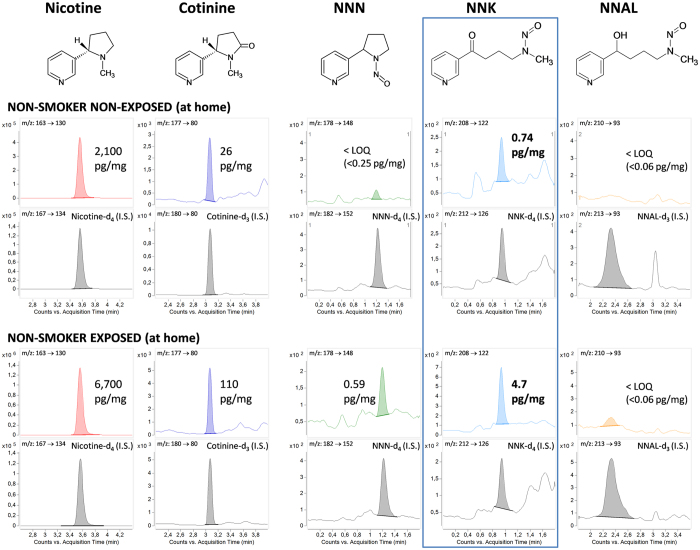
Chromatograms of the analysis of hair samples from a representative exposed non-smoker (living with at least one smoker) and a non-smoker living in a smoke-free home. Chromatograms are given for the individual analytes (nicotine, cotinine, NNN, NNK and NNAL) and their respective deuterated internal standards (I.S.). NNK, the most abundant TSNA in hair samples, is highlighted.

Nicotine and cotinine concentrations spanned over a wide range with medians of 1,160 pg/mg and 37 pg/mg respectively and correlated well (Spearman’s coeff. rsp = 0.779, p < 0.001). Table 2 gives the results segregated by declared exposure the declaration of being exposed at home (living with a smoker or in a smoke-free home). The analysis of TSNAs showed undetectable or not quantifiable concentrations of NNAL in all samples. However NNK, its precursor and potent carcinogen, was detected in concentrations ranging 0.1–7.8 pg/mg. NNN could also be detected in samples from some of the most exposed individuals. Figure 1 shows representative chromatograms of the analysis of a self-declared exposed individual and a person living in a smoke-free home.

**Figure 2:**
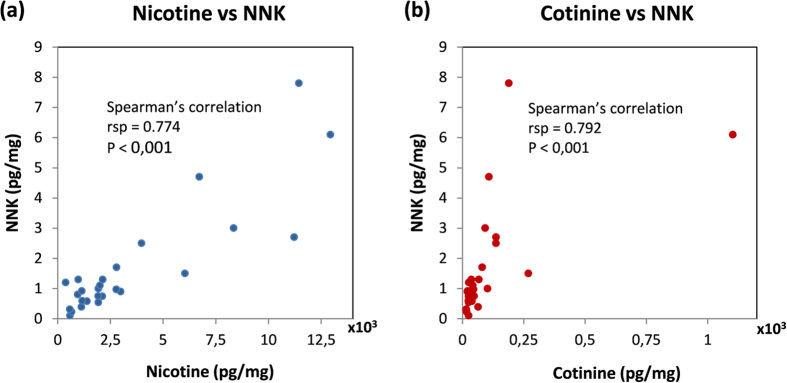
Plots showing the correlation between NNK and nicotine (**a**) or cotinine (**b**) found in hair samples from 48 non-smokers participating in the study.

NNK correlates very significantly with nicotine concentrations (Spearman’s coeff. rsp = 0.774, p < 0.001) and also with cotinine (Pearson’s coeff. r = 0.792, p < 0.001) (Fig. 2). Interestingly, no correlation was found between NNN and any of the other analytes, although probably the total number of samples for which a concentration of NNN could be measured does not allow obtaining definitive conclusions. The NNK/nicotine ratio was found to show a wide variation, even with this good correlation. Several samples with similar nicotine concentrations showed very different NNK values (See Fig. 2a). The overall median of the ratio NNK /nicotine was 0.49·10^−3^ with an interquartile range 0.35·10^−3^–0.64·10^−3^. When the calculation is segregated by exposed or not exposed (at home) values show a slight trend towards higher values of the ratio for exposed individuals (Table 2).

## Discussion

The evaluation of health risks linked with the exposure to SHS, and particularly those associated with different types of cancer, has led research efforts to the study of TSNAs. With the implementation of smoke-free laws in many countries, active efforts have been directed towards monitoring exposure in non-smokers. The analysis of hair samples gives a good estimate of averaged exposure over a period of more than one month. Nicotine and cotinine concentrations were found to be consistent with those already reported in similar populations^20^. Exposure to tobacco specific carcinogens (i.e. TSNAs) has been regularly measured by quantifying total NNAL in urine samples, although it is known to represent less than 20% of the NNK dose intake in smokeless tobacco users^47^. However, NNAL has been shown in low concentrations in toenails or hair from smokers, and it has shown to be undetectable in samples from non-smokers^41^,^42^. NNN had been detected in toenails of non-smokers with a mean concentration of 0.062 pg/mg and it correlated with cotinine concentrations^43^. This study shows for the first time that NNK is the most abundant TSNA present in hair and it can be detected in samples from non-smokers in concentrations much higher than any other nitrosamine. The analysis of other biological matrices like urine shows that NNK is absent after extensive metabolism and NNAL is chosen as the main NNK metabolic marker. However, the situation in hair has proven to be completely different. Analogous to what happens with nicotine vs cotinine, the concentrations of the precursor NNK showed to be much higher than those of its metabolic analogue NNAL which is consistently undetectable in samples from non-smokers. Concentrations of NNK in samples from non-smokers were up to 7.8 pg/mg, with an overall mean of 1.7 pg/mg. The availability of low amounts of hair for some subjects (e.g. down to 2.4 mg) and the real lack of exposure to SHS of some others, accounts for those samples without quantifiable amounts of NNK. NNAL was always below the limit of quantification (0.06 pg/mg). Conversely, reported concentrations of NNAL in hair samples from heavy smokers never went above 0.7 pg/mg, while in toenails mean concentrations were 0.41 pg/mg. Sample amounts between 50 and 150 mg were used^41^,^42^. Nicotine, cotinine and NNK values were significantly higher in those subjects declaring being exposed. The number of samples where NNN could be quantified precluded this evaluation, although the values showed a similar trend. NNK concentrations correlate very significantly with nicotine and cotinine. When volunteers were grouped by their degree of exposure to SHS^19^, significant differences were found, showing the goodness of the biomarkers. Interestingly, NNN could also be quantified in samples from the non-smokers most exposed to SHS, but the concentrations did not correlate with any of the other analytes. No correlation was found between salivary cotinine concentrations^19^ and hair cotinine (Pearson’s coeff. r = 0.182, p = 0.289), NNK (Pearson’s coeff. r = 0.102, p = 0.621) or NNN (Pearson’s coeff. r = −0.122, p = 0.754). However, a weak correlation could be found between salivary cotinine and hair nicotine (Pearson’s coeff. r = 0.344, p = 0.019). The mechanism of incorporation of the substance into hair and probably the whole metabolic fate of NNN may play an important role. NNAL/cotinine ratios in urine samples have shown differences between active and passive smokers^28^,^36^,^37^. A NNK/nicotine ratio in hair is a very promising new marker and its values should be compared with those obtained from smokers in order to confirm if a significant increase in the relative NNK exposure with respect to nicotine could be detected in passive smokers.

The method developed has proven to be a reliable tool for analyzing nicotine, cotinine, NNN, NNK, NNAL, in human hair samples. Considering the important differences between individual hair samples (colour, cosmetic treatments, age of the donors, etc.) and the unavoidable varying matrix composition, it became obvious that the use of calibration curves in a particular hair sample did not solve those problems. Besides, a reliable source of real blank hair samples is not easy to find, bearing in mind the ubiquity of tobacco smoke in our environment. Accordingly, we followed a validation strategy^48^ where calibration curves are prepared both in matrix and in the absence of matrix (taking the standards through the whole sample preparation and analysis process as real samples) but QC samples are prepared in matrix and quantified against both curves. Limits of quantification are comparable to previous publications. LOQs depend mainly on the amount of sample available and to a minor extent on the individual matrix effect, as it might not equally affect signal and noise. The method was developed using 20 mg of hair only, considering that the amount of sample is frequently a limiting factor.

Nicotine in hair is a very good biomarker of long-term exposure to SHS and it is the easiest to detect^48^. However, these results show that NNK exposure can be reliably measured and its ratio to nicotine might vary from individual to individual. Therefore, monitoring NNK in hair should be a much better marker of individual carcinogenic risk, especially among non-smoker population, irregularly exposed to SHS.

This study has several limitations that is worth mentioning. As in any hair analysis, the hair composition (e.g. colour of the hair, cosmetic treatments, etc.) might have an impact on the final concentrations found. The number of samples analysed has to be increased in order to better correlate exposure or smoking habits with these TSNAs. In particular, samples from recreational and heavy smokers need to be analysed for this reason.

## Methods

### Safety Hazards

NNK, NNN and NNAL are carcinogenics and mutagenics and should be handled with extreme care.

### Chemicals

(−)-Nicotine and (−)-cotinine 1.0 mg/mL standard solutions in methanol as well as HPLC grade formic acid were purchased from Fluka-Sigma-Aldrich (Madrid, Spain). ( ± )-nicotine-d_4_ (2,4,5,6-tetradeutero-3-(1-methylpyrrolidin-2-yl)-pyridine) 100 μg/mL solution in acetonitrile and ( ± )-cotinine-d_3_ (5-(3-pyridinyl)-1-trideuterometyl-2-pyrrolidinone) 1.0 mg/mL in methanol, were purchased from Cerilliant Corp (Round Rock, Texas, USA). (±)-*N*′-nitroso-nornicotine (NNN), (±)-NNN-d_4_ (2,4,5,6-tetradeutero-(5-(1-nitroso-2-pyrrolidinyl))-pyridine), 4-(methylnitrosamino)-1-(3-pyridyl)-1-butanone (NNK), NNK-d_4_ (4-(methylnitrosamino)-1-(2,4,5,6-tetradeutero-3-pyridyl)-1-butanone, (±)-4-(methylnitrosamino)-1-(3-pyridyl)-1-butanol (NNAL) and (±)-NNAL-d_3_ (4-(trideuteromethyl-nitrosamino)-1-(3-pyridyl)-1-butanol were purchased from Toronto Research Chemicals (Ontario, Canada). HPLC grade methanol, acetonitrile and 2-propanol, as well as analytical grade sodium hydroxide, potassium chloride, 25% ammonia solution and 37% fuming hydrochloric acid were obtained from Merck Millipore (Darmstadt, Germany). HPLC grade dichloromethane was purchased from Scharlau (Barcelona, Spain). Ultrapure water was produced using a Millipore Milli-Q water purification system. Nitrogen was obtained from a central high flow permanent supply using a liquid nitrogen bulk tank (99.5%, Praxair, Spain). Sample processing tubes were KIMAX 16 × 125 mm screw cap glass borosilicate tubes from Kimble Chase (Queretaro, Mexico).

### Subjects

48 non-smokers of both sexes were recruited from a cross-sectional study to evaluate the impact of the Spanish smoke-free law 28/2005 on the exposure to secondhand smoke of the adult population of the city of Barcelona, Spain^19^,^49^. The research and ethics committee of Bellvitge University Hospital (L’Hospitalet de Llobregat, Barcelona, Spain) approved the study protocol, and all participants signed an informed consent. The study was performed in accordance with the Declaration of Helsinki ethical principles for medical research involving human subjects. Data was treated in compliance with the Spanish Law of Personal Data Protection (Law 15/1999). Participants were asked if they were exposed to SHS. Hair samples, cut close to the scalp, were collected from the vertex posterior where possible. They were kept in individual envelopes at room temperature with both ends (proximal and distal) identified.

### Sample preparation

The segment of hair to be analysed (i.e. 1.5 cm of the proximal end, close to the scalp) was first washed three times with CH_2_Cl_2_ by sonication for 10 min^38^. After drying at a temperature below 40°C, samples were put in flat bottomed plastic tubes (50 × 16 mm), finely cut with scissors and kept at room temperature until analysis. The sample preparation procedure is similar to other previously published with some modifications^50^,^51^. An amount of ca. 20 mg was weighed in a sample processing tube and 50 μL of internal standard (IS) solution (10 ng nicotine-d_4_, 2 ng cotinine-d_3,_ 20 pg NNN-d_4_, 20 pg NNK-d_4_ and 4 pg NNAL-d_3_) were added. The mixture was digested for 30 min at 80 °C in 1 mL 1 M NaOH, 2 M KCl aqueous solution and then allowed to cool to room temperature^38^. The resulting digested samples were extracted with 5mL CH_2_Cl_2_ and 5 mL CH_2_Cl_2_:iPrOH (75:25) consecutively by rocking mixing for 15 min at a frequency of 50 min^−1^ and centrifuging at 2,026 g for 10 min. The combined organic phases were back-extracted with 1 mL 0.5 M HCl in another sample processing tube. The aqueous phase was made alkaline with 1 mL 1M NaOH, 2M KCl and extracted again with 4 mL CH_2_Cl_2_ and 4 mL CH_2_Cl_2_:iPrOH (75:25) consecutively. The organic phases were combined and, after addition of 100 μL 25 mM HCl in MeOH, concentrated to dryness under a gentle N_2_ stream. Dry extracts were re-dissolved in 100 μL ACN/MeOH (95:5) and transferred to an injection vial with a small volume insert and kept in the instrument autosampler at 4 °C. The suitability of the use of calibration curves in the absence of matrix was tested during method validation. Calibration curves and quality controls (low medium and high) were prepared in 20 mg blank hair and back-calculated using the calibration curve prepared in absence of matrix to test for the adequacy of the method. Precision and accuracy of the quantifications were required to be within ± 15% along the whole calibration range (including medium and high QCs) and within ± 20% for the LLOQ (and low QC). The impact of the amount of hair analysed on the quantification was tested by homogenizing a hair sample known to contain detectable concentrations of the analytes, and analyzing amounts of 5, 10, 20 and 50 mg. Calibration curves were prepared covering a double range during method validation, one for non-smoker samples and another for potentially high concentrations. Ranges were 25–2,500 pg/mg (or up to 500 ng/mg) for nicotine, 2.5–2,500 pg/mg (or up to 500 ng/mg) for cotinine, 0.25–2.5 pg/mg (or up to 10 pg/mg) for NNN, 0.10–2.0 pg/mg (or up to 50 pg/mg for NNK) and 0.063–0.40 pg/mg (or up to 6.3 pg/mg) for NNAL.

### Liquid chromatography-tandem mass spectrometry

Instrumental analyses were performed on an Agilent Technologies LC 1290 Infinity HPLC system connected to an Agilent 6490 triple quadrupole mass spectrometer, through an iFunnel ionization source working in positive ionization mode. The chromatographic separation was achieved using a normal phase chromatographic column Cogent Diamond Hydride column 100 mm long, 2.1 mm I.D. 120 Å pore, 4.0 μm particle size. The auto-sampler tray temperature was kept at 4 °C. The needle of the injector was externally rinsed with MeOH for 15 seconds prior to each injection. The mobile phase passed through the needle for the whole duration of the chromatographic run. The column temperature was kept at 35 °C and the volume injected was 10 μL.

Chromatographic separation was achieved using a binary gradient of (A) 100 mM aqueous ammonium formate solution taken to pH 3 with formic acid and (B) acetonitrile, at a flow rate of 0.6 mL/min. The gradient was: isocratic at 1% A during 1.5 min; increased linearly up to 5% A in 0.5 min; increased linearly up to 50% A in 0.5 min; kept at 50% A for 1.5 min; return to initial conditions in 1 min. Total chromatographic time 5 min. Acquisition was performed in multiple reaction monitoring (MRM) mode. iFunnel source conditions were as follows: capillary voltage (positive), 3000 V; desolvation gas temperature, 100 °C; drying gas flow, 12 L/min; nebulizer, 35 psi; sheath gas heater, 275 °C; sheath gas flow, 12 L/min; charging voltage, 750 V. High-purity nitrogen (99,999%, Abello-Linde, Spain) was used as collision gas. As nebulizer and drying gas, nitrogen was obtained from a central high flow permanent supply using a liquid nitrogen bulk tank (99.5%, Praxair, Spain).

MS/MS parameters were optimized by injecting 10 μL of 10 ng/mL individual standard solutions in acetonitrile. Fragmentor voltage at 380 V, cell accelerator voltage at 4 V and dwell time 58 ms were used in all cases. Collision energy was optimized for each compound and fragments being between 9 and 49 V for all transitions. The MRM transitions for quantification and identification were respectively: *m*/*z* 163 to 130 and 117 for nicotine and *m/z* 167 to 134 and 121 for nicotine-d_4_; *m*/*z* 177 to 80 and 98 for cotinine and m/z 180 to 80 and 53 for cotinine-d_3_; *m*/*z* 178 to 148 and 119 for NNN and m/z 182 to 152 and 124 for NNN-d_4_; *m*/*z* 208 to 122 and 79 for NNK and *m*/*z* 212 to 126 and 83 for NNK-d_4_; *m*/*z* 210 to 93 and 108 for NNAL and *m*/*z* 213 to 93 and 108 for NNAL-d_3_. All data were acquired and processed using MassHunter Quantitative Analysis v B.06.00 software.

### Statistical analyses

We described the concentrations of biomarkers using median (interquartile range (IQR)) and mean (standard error, SE) according to the self-reporting SHS exposure at home. We compared the concentrations of biomarkers according to the self-reporting SHS exposure at home using the Mann Whitney U-test for two independent samples. Given the skewed distribution of biomarkers, we used Spearman’s rank correlation coefficient (rsp) to assess the association between biomarkers.

For all analyses, we used PASW Statistics 18, Release 18.0.0 2009 (SPSS, Inc., Chicago, IL).”

## Additional Information

**How to cite this article**: Pérez-Ortuño, R. *et al*. Evaluation of tobacco specific nitrosamines exposure by quantification of 4-(methylnitrosamino)-1-(3-pyridyl)-1-butanone (NNK) in human hair of non-smokers. *Sci. Rep.* **6**, 25043; doi: 10.1038/srep25043 (2016).

## Figures and Tables

**Table 1:** Precision (%CV) and accuracy (%err) intra-assay values obtained for the quality control samples (QC) analysed in parallel with the “non-smoker” calibration curves.

QC	Nicotine			Cotinine			NNN			NNK			NNAL		
	*Conc. (pg/mg)*	*%CV*	*%err*	*Conc. (pg/mg)*	*%CV*	*%err*	*Conc. (pg/mg)*	*%CV*	*%err*	*Conc. (pg/mg)*	*%CV*	*%err*	*Conc. (pg/mg)*	*%CV*	*%err*
Low	**30**	7.0	11	**3**	19	−13	**0.3**	4.1	−0.8	**0.13**	9.0	6.6	**0.075**	2.9	19
Medium	**750**	0.6	8.9	**750**	9.2	0.5	**0.75**	3.6	−0.6	**0.75**	3.6	−3.2	**0.15**	2.5	−1.4
High	**2,000**	1.9	12	**2,000**	5.7	8.7	**2.0**	2.3	0.7	**1.7**	4.9	8.4	**0.35**	2.1	0.6

**Table 2:** Concentration of the analytes found in hair samples from 48 non-smokers segregated by their self-declaration of living with at least one smoker or in a smoke-free home.

Group	Nicotine	Cotinine	NNN	NNK	NNAL
Exposed at home (N = 24)					
Median (IQR) [pg/mg]	2,040 (1,200; 4,650)	49 (26; 106)	0.54 (0.29; 0.60)	1.3 (0.92; 2.7)	–
Mean (SE) [pg/mg]	3,320 (664)	74 (14)	0.54 (0.067)	2.1 (0.40)	–
N (quantifiable)	24	21	7	15	0
Non-exposed at home (N = 24)					
Median (IQR) [pg/mg]	623 (221; 1,160)	26 (16; 43)	0.41 (−)	0.74 (0.31; 1.1)	–
Mean (SE) [pg/mg]	1,330 (527)	95 (55)	0.41 (0.027)	1. 1 (0.34)	–
N (quantifiable)	24	16	2	11	0
p (2-tailed) (Mann-Whitney)	0.001	0.030	–	0.018	–

Median (interquartile range (IQR)) and mean (standard error, SE) are given for each group.
